# Book Review: Basic Counseling Skills: A Helper's Manual

**DOI:** 10.3389/fpsyg.2018.02031

**Published:** 2018-10-23

**Authors:** Parwinder Singh

**Affiliations:** Humanities and Social Sciences, Indian Institute of Technology Ropar, Rupnagar, India

**Keywords:** counseling skills, Richard nelson-jones, critical review, counselor, practitioners

For an effective outcome of the psychological counseling, the therapist must be knowledgeable about various dynamics of human nature and be skillful enough to apply his/her knowledge in one to one interaction with the client. Learning of basic counseling skills is an integral part of the counselor's training. The need to transform young psychologists into effective counselors had been well understood by many scholars, but it was Richard Nelson-Jones who provided the trainees with material that renovated them into actual effective helpers. Richard Nelson-Jones, a Counselor and Counseling Psychology Trainer has 37 books to his credit, most of which are intended to empower helpers with effective counseling skills, e.g., Practical Counseling And Helping Skills: Text And Activities For The Life Skills Counseling Model (2005), to name the one. The latest contribution of Richard Nelson-Jones, i.e., Basic Counseling Skills: A Helper's Manual (Third Edition) also has the same intention. The first edition of the book was published in 2003, and the latest came in 2012. In comparison to previous editions, the third edition of this book presents the more practical introduction of necessary counseling skills with the help of vivid case example and experimental activities. Therapeutic dialogues have also been incorporated with more preciseness.

The book is intended to support the training and practice of counseling psychologists and provides a concise and to-the-point summary of counseling skills. The author divided the book into 28 brief chapters which are clubbed into three parts. Part one provides an introductory discussion of Counseling and its processes. Author has tried to clarify most of the doubts which the beginners may face while entering into this profession. In part two, the author introduced a wide range of basic counseling skills, presented separately in 18 chapters along with numerous examples and activities. The specific counseling skills, preferred by the author over others are understanding the internal frame of reference, paraphrasing and reflecting feelings, asking questions, monitoring the process, feedback offering, self-disclosure, facilitating problem-solving, relaxation procedure and terminating sessions. Apart from the introduction of the profession and basic counseling skills, the author discussed other very important aspects of the counseling profession, e.g. ethical issues & dilemmas. Cultural and gender considerations have also been talked about in the concluding chapters. The author suggested young counselors observe and listen to the demonstrations of skilled counselors and helpers and keep them updated by reading the latest books and relevant journals. Its thorough, step-by-step guidance to the subject and presentation of the working of each stage of the helping process make it unique among books available on similar issues.

Critically reviewing the book resulted in many positive and negative observations. The book looks attractive with a very soothing color combination of its cover page. Apart from the introduction of the author and preface, another section contains “Praise for the Book” by Professor Michael Carroll from the University of Bristol which add the curiosity among readers to explore the book. The language used in the book is not at all technical and the message is conveyed in a very simple and precise manner that is appropriate for both professionals and non-professionals. The direct conversational tone in writing makes the readers feel that the author is talking directly to them which may increase the concentration span of the reader. Each chapter starts with a basic paragraph about the topic, and then the main theme is discussed along with precise and appropriate examples provided into separate boxes which make it more attractive. All the chapters are concluded with very useful activities which make sure that the reader acquires the required skills and practice it in the simulated or real-life setting. Overall the book serves what it is supposed to serve.

Like any other publication, this book is also not free from shortcomings or limitations. The content of the book is not comprehensive with less elaboration on the skills which is good for non-professionals who want to go through the book at a shallow level, but for academicians and professionals, it does not provide sufficient exposure of the world of counselors. A little more elaboration on each skill could make this book more effective. To make it simple and precise, the author had compromised with the quality and quantity of the content. To make it less technical, the author did not include any reference throughout the text of the book which makes it less authentic and shows author's more subjective assertions regarding counseling skills and it seems to the readers that the author has little or no objective evidence of the effectiveness of the skills mentioned in the book. Moreover, the skills mentioned in the book are not shown to be supported by theoretical models without which the effectiveness of the skills is doubtful. The books might be a complete manual if the researches work conducted so far in the area of counseling skills were included in the text along with its references.

Overall, the book effectively caters to the needs of young counselors or practitioners. Throughout, the information is presented in a precise manner that will prove very useful for both beginners and the more experienced practitioner to refer to in order to keep up and develop their counseling skills. Taking the price of the book and all other pros and cons into consideration, I recommend this book as a reference book to all in the counseling profession.

## Author contributions

The author confirms being the sole contributor of this work and has approved it for publication.

### Conflict of interest statement

The author declares that the research was conducted in the absence of any commercial or financial relationships that could be construed as a potential conflict of interest.

